# Books and Authors

**Published:** 1906-01

**Authors:** 


### BOOKS AND AUTHORS.

---

**Textbook on the Practice of Medicine.** By **James Magoffin French,** M.D., Formerly Lecturer on the Theory and Practice of Medicine, Medical College of Ohio. Second revised edition. Octavo, pp. 799. Illustrated. New York: William Wood & Co. 1905. (Price, $4.00.)

We are not surprised to see a second edition of this excellent treatise called for so soon. In this Journal, in March, 1904, we wrote concerning the first edition as follows:

> Its make-up and general form are excellent; it is terse even to conciseness without being elementary; it deals with only the practical. leaving the theoretical behind; it contains the most modern thought and records the most recent discoveries; it omits case reports and instead deals with more useful material, thus economising space and preserving the patience of the reader; finally, it is illustrated with a few well selected and excellently executed plates and engravings that give force and impact to the text. For these and other reasons, not necessary here to enumerate, we are of the opinion that this work will take an important place in medical literature.

Now, two years since the original issue of the book, we are prepared to reaffirm this opinion. The second edition presents some changes as a matter of course, principally regarding acute infectious diseases and animal parasites, while some of the chapters have been rearranged and partially rewritten. New methods of treatment, too, receive appropriate reference, but the size and general appearance of the book remains unchanged. It is a treatise that will make an addition of value to the library of any physician who may not already possess it.

**A Textbook of Physiology, Normal and Pathological.** For Students and Practitioners of Medicine. By **Winfield S. Hall**, Ph.D., M.D., (Leipzig), Professor of Physiology, Northwestern University Medical School, Chicago. Second edition, revised and enlarged. Octavo, 795 pages, with 339 engravings and three full-page colored plates. Philadelphia and New York: Lea Brothers & Co. 1905. (Cloth, $4.00 net.)

This clever book by a clever author is entitled to receive a hearty welcome in its second edition, by every student as well as every teacher of physiology. In the first place the author's method of teaching physiology is based on sound principles. He recognises it as an experimental and superstructural science, built upon anatomy, chemistry, and physics and has summarised in the introduction those principles of physics and chemistry which have general application. Besides, he has prefixed to each chapter an abstract of the facts drawn from all three of the basic sciences which are to be applied in the succeeding text.

This edition has been carefully revised, considerably enlarged, and modified in such a manner as to meet the existing conditions of physiologic science. To the chapters on circulation and blood, respiration, digestion, metabolism, and excretion have been added sub-chapters on pathologic physiology. Clinicians and pathologists have been drawn upon for aid in preparing the material on pathologic physiology.

Hall's treatment of the physiology of the nervous system, of the special senses, and of reproduction, is deserving of particular commendation. He discusses the pathologic physiology of the former two within the body of the chapters pertaining to them.

There are many special features of this book that merit comment, but we deem it unnecessary to enter into detail with a work so well known; yet we cannot refrain from mention of the sub-chapter on the pathologic physiology of excretion, which is a gem.

The illustrations are far above the common order in works of this character, and the book as a whole is deserving of the highest commendation.

**A Practical Treatise on Fractures and Dislocations.** By **Lewis A. Stimson**, B.A., M.D., Professor of Surgery in Cornell University Medical College, New York; Surgeon to the New York and Hudson Street Hospitals. Fourth edition, thoroughly revised. Octavo, 844 pages with 331 engravings and 46 plates. Philadelphia and New York: Lea Brothers & Co. 1905 (Cloth, $5.00; leather, $6.00; half morocco, $6.50 net prices.)

Since the disappearance of Hamilton's classic treatise on these subjects, Stimson has become the recognised standard authority on fractures and dislocations. He is an experienced and capable teacher, whose counsel may be followed in safety by every physician who is called upon to minister to the injuries with which this treatise deals.

Every surgeon is familiar with the fact that fractures tax his skill and energy beyond that of any other branch of his art. The laity demand immediate service in these injuries and the surgeon must be ready always to render it. Stimson in his first nine chapters deals with general questions relating to the pathology, symptoms, diagnosis, delayed union, etiology, prognosis and treatment of fractures. These chapters show the astute surgeon in every line and should be studiously examined by every junior physician who expects to treat fractures.

This edition, the fourth, has been revised and considerably enlarged, to embrace the latest concerning the treatment of fractures in and near joints, and the systematic use of *x*-rays, many skiographs having been inserted. The operative treatment of old dislocations, too, has received additional emphasis by the new material added to this topic.

There is small need of taking up valuable time with minute details, in discussing at length the several methods adopted by the author in treating these traumatisms; it is quite sufficient that the latest and best method has been adopted wherever there has been improvement and when the old is best that is adhered to. The work is a credit to American surgery and is quite equal to any similiar treatise anywhere in the world.

**American Edition of Nothnagel's Practice.** Diseases of the Kidney, of the Spleen, and Hemorrhagic Diseases. By **Drs. H. Senator,** and **M. Litten,** Berlin. Edited with additions, by James B. Herrick, M.D., Professor of Medicine in Rush Medical College, Chicago. Octavo, 816 pages, illustrated. Philadelphia and London: W. B. Saunders & Co. 1905. (Cloth, $5.00 net; half morocco, $6.00 net.)

Senator is an acknowledged authority on diseases of the kidney, hence his work finds an appropriate place in the Nothnagel series. While the literature of renal disease has grown to large proportions of late, in this volume Senator has presented a digest of all that is important pertaining to it; and he also has presented his own views in a most entertaining and instructive manner.

The author remains silent upon the surgical treatment of nephritis but the editor, Herrick, enters a protest against the procedure; at least, he does not think the statistics are convincing that chronic interstitial nephritis has been cured by surgical intervention.

The other topics presented in this volume,—diseases of the spleen and hemorrhagic diseases, — are written by Professor Litten, also of the University of Berlin, and are exceedingly well handled. Palpation is the chief reliance in diagnosis, and detailed directions are given for its employment.

Scurvy and its related conditions,—hemophilia and morbus maculosus Werlhofi, are described under hemorrhagic diseases. The same careful expression of the latest scientific thought pervades this section of the book as diffuses the others, and the whole constitutes a most valuable addition to the literature of medicine.

**International Clinics.** A Quarterly of Illustrated Clinical Lectures and especially prepared articles on Treatment, Medicine, Surgery, Neurology, Pediatrics, Obstetrics, Gynecology, Orthopedics, Pathology, Dermatology, Opthamology, Otology Rhinology, Laryngology, Hygiene and other topics of interest to students and practitioners. By leading members of the medical profession throughout the world. Edited by **A. O. J. Kelly,** A.M., M.D., Philadelphia. Volume II. Fifteenth series. 1905. Philadelphia and London: J. B. Lippincott Co. (Cloth, $2.00)

These volumes improve as they increase in number, the present book being one of the very best yet issued. It contains five articles on treatment; five on medicine; eight on surgery; one on gynecology; one on ophthalmology; one on rhinology; one on physiology; and one on pathology.

The articles for the most part are of a practical character and will serve as aids to the younger practitioner at the bedside. Dr. Palmer's contribution to the rational therapy of uterine displacements is one of the best yet presented on the subject. Almost any one of the monographs is worth more than the price of the book.

**A Textbook on the Practice of Gynecology.** For Practitioners and Students. By **W. Easterly Ashton,** M.D., LL.D., Professor of Gynecology in the Medico-Chirurgical College of Philadelphia. Philadelphia and London: W. B. Saunders & Co. 1905. (Cloth, $6.50 net; half morocco, $7.50 net.)

That in a multiplicity of counsel there is wisdom, is an axiom that needs no elaboration. Many excellent textbooks and works on gynecology have been presented to the profession within the preceeding twelve or fifteen years, each having special features not to say merits, and Ashton's furnishes no exception to the rule. If it does not contain startling novelty, it yet states many well established truths in a forceful way, and in a manner to attract attention.

The chapter on indoor exercise deserves careful study; it is new in a work on gynecology. The chapter on the vulva is one of the very best in the book; it discuses topics often omitted. The urinary tract from the meatus to the kidney is well handled, especially as regards the ureters. The gynecologist who is also a bacteriologist will like this book because it gives the necessary instructions regarding collecting secretions and as to the preservation of specimens.

The illustrations are numerous,—1046, in a book of 1041 reading pages,—and are line drawings instead of half-tones and etchings, as is usual in such a work. The Sims position is pictured left handed, which does not matter much for the experienced gynecologist, but is liable to mislead the novice.

An excellent index brings to an end a contribution to gynecological literature that will naturally find a place in the library of every teacher and practitioner of this branch ot medical science.

**The Diagnosis of Diseases of Women.** For Students and Practitioners. By **Palmer Findley,** B.S., M.D., Assistant Professor of Obstetrics and Gynecology, Rush Medical College, Chicago. Octavo, 588 pages, illustrated with 222 engravings and 59 plates in color and monochrome. Philadelphia and New York: Lea
The early exhaustion of the first edition of Findley's excellent
Brothers & Co., 1905. (Cloth, $4.75; leather, $5.75, net prices.)

The early exhaustion of the first edition of Findley's excellent work has offered opportunity to make important additions to this issue, and the author has made the most of the occasion. Especially has he presented additions in relation to the differential diagnosis in diseases of the genito-urinary organs; in disputed subjects, like cystic degeneration of the ovaries chorioepithelioma malignum, and in diseases of the ureters and kidneys. New chapters, too, appear on examination of the blood, and bacteriological examinations. Many new illustrations and colored plates have been added, most of which are original, while the text has been increased by about one hundred pages. This work is needed by everyone who practises gynecology, as a complement to the remander of his literature on the subject.

It is such a book as this that scores a credit mark to the literature of medicine. Overcrowded as its library is, there is always room in the front shelves for a work that advances the science and art of medicine in the manner that this book does.

**Progressive Medicine, Vol. III, September, 1905.** A Quarterly Digest of Advances, Discoveries and Improvements in the Medical and Surgical Sciences. Edited by **Hobart Amory Hare,** M.D., Professor of Theraputics and Materia Medica in the Jefferson Medical College of Philadelphia. Octavo, 298 pages, with 22 engravings. Per annum, in four cloth-bound volumes, $9.00; in paper binding, $6.00. Carriage paid to any address. Lea Brothers & Co., Publishers, Philadelphia and New York

The subdivisions of medicine discussed in this number are: (1) diseases of the thorax and its viscera, by Professor Wm. Ewart; (2) dermatology and syphilis, by Gottheil; (3) diseases of the nervous system, by Wm. G. Spiller, and (4) obstetrics, by Richard C. Norris.

The tuberculosis topic receives elaborate treatment at the hands of Ewart; some unusual conditions and new methods of treatment in dermatology and syphilis are recorded by Gottheil; a miscellaneous group of nervous diseases of great interest is presented by Spiller; a number of important questions in obstetrics is offered by Norris, and an excellent index is added.

This digest appeals with cogency to every physician who would keep himself well informed as to medical progress, and this particular number is an excellent specimen of the work that is being done by editor, contributors and publishers.

**Atlas and Textbook of Topographic and Applied Anatomy.** By **Professor O. Schultze,** Würzburg. Edited with additions by George D. Stewart, M.D., Professor of Anatomy and Clinical Surgery, University and Bellevue Hospital Medical College, New York. Quarto, 187 pages, containing 22 colored lithographic plates, and 89 text-cuts, 60 in colors. Philadelphia and London: W. B. Saunders & Co. 1905. (Cloth, $5.50 net.)

Regional anatomy is an essential in the studies of the surgeon, and no more helpful textbook on this subject has been issued in recent years, than the one with the foregoing title. It is also a book that may be referred to and even studied by the general practitioner of medicine.

The colored lithographic plates are almost the perfection of anatomic illustration, while the figures in the text, which number nearly ninety, are of conspicuous excellence. Schultze's dictum, "Think anatomically if you wish to become a physician," is a most fitting maxim for the undergraduate. The entire make-up of this splendid work, in type face, page, illustration, paper and binding is beyond criticism, and it is a book that distinctly adds to the anatomic armamentarium of the physician.

**A Manual of Practical Hygiene.** By **Charles Harrington,** M.D., Assistant Professor of Hygiene in Harvard University Medical School, Boston. Third edition, revised. Octavo 793 pages with 118 engravings and 12 plates. Philadelphia and New York: Lea Brothers & Co. 1905. (Cloth, $4.25.)

Professor Harrington is the acknowledged authority on hygiene as related to both public and private life. His official relations to the state department of health of Massachusetts, and his experience as a teacher of hygiene in Harvard University entitles him to speak from the chair on all topics that, for the sake of convenience, are grouped under the general name of hygiene which also includes sanitation.

This work, according to the author's modest statement, is designed especially for students, physicians and health officers. In our view, however, it has a still wider range, and is adapted to the needs of every physician who has any conception of the propriety of a thorough understanding of preventive medicine. The author has made such changes in this edition as were necessary to bring the work forward to the present date, thus making one of the best manuals on hygiene that has yet been presented.

**Handbook of Anatomy.** A Complete Compend of Anatomy, including the Anatomy of the Viscera and Numerous Tables. By **James K. Young,** M.D., Professor of Orthopedic Surgery, Philadelphia Polyclinic. Second edition, revised and enlarged. With 171 engravings some in colors. Crown octavo, 404 pages. Philadelphia: F. A. Davis Company. 1905. (Flexible cloth, rounded corners, $1.50 net.)

The author of this anatomical handbook is one of the first anatomists of the period; he is, it is true, an orthopedic surgeon, but before he became an expert orthopedist, which he is acknowledged to be, he was of necessity an anatomist.

Viewed from any standpoint, this handbook must be awarded praise. It rises to the level of its aim; it claims to be a complete compend of anatomy and it is a most complete synopsis of that subject. It is useful for a student in the anatomical laboratory, and for a physician who needs to refresh his memory upon forgotten or beclouded portions of human anatomy.

In this second edition the author has revised the text where essential, changed and increased the illustrations to a considerable extent, and modfied or improved the sections wherever necessary, even having rewritten some of them. The book is considerably larger than formerly, and is one of the best anatomical manuals in print.

**Ophthalmic Neuromyology.** A Study of the Normal and Abnormal Actions of the Ocular Muscles from the Brain Side of the Question. By **G. C. Savage,** M.D., Professor of Ophthalmology in the Medical Department of Vanderbilt University. Small 8vo, pp. 221. Illustrated. Nashville: Keelin-Williams Printing Co. 1905.

The author of this "study" is an ophthalmologist of large experience and his writings are entitled weight. The nervo-muscular mechanism of the eye is a complex arrangement, not easily understood by the novitiate, but this book should make the study easier; at least that is one of the objects of the author, and we believe he has succeeded in accomplishing that part of his purpose at least.

The work is well illustrated and is published and copyrighted by the author himself, who should derive substantial benefit from his labor. We commend the book to every physician who has any penchant for the study of the ocular mechanism.

**A Textbook of Pathology and Pathological Anatomy.** By **T. Henry Green,** M.D., F.R.C.P., Consulting Physician to Charing Cross Hospital, London. New (tenth) edition. Thoroughly revised by Cecil Bosanquet, A.M., M.D., F.R.C.P., Assistant Physician to Charing Cross Hospital. Octavo, 606 pages, 348 engravings and a colored plate. Philadelphia and New York: Lea Brothers & Co., Publishers. 1905 (Cloth, $2.75 net.)

Green's pathology has been too long before the profession to need more than a formal announcement that a new edition is ready for student and practitioner. This has always been a popular textbook because of its clearness of statement, its simplicity of style, and its perfection of material. It has kept pace with the transformations through which pathology has passed and each edition has offered something new. This one has been revised in text and numerous additions have been made, especially in the fields of animal parasitology and immunity to infectious diseases. Some of the older illustations have given way to newer figures, and several new ones have been added, making the work still one of the most desirable on the subject in existence.

**Physical Diagnosis.** By **Richard C. Cabot,** M.D., Instructor in Medicine in Harvard University. Third edition, revised and enlarged. Small octavo, pp. xxii.-577. Illustrated. New York: William Wood & Company. 1905.

This valuable book is again presented for the inspection of the profession. The previous editions have met with favor, and it cannot be doubted that this one will receive even more commendation than its predecessors. It is a personal book, hence far more valuable than one which presents everything that "has been recommended." Cabot writes of what he knows to be valuable as an aid to diagnosis, not of what somebody has told him is good. He describes even in detail the small things which often prove of great assistance. The illustrations, consisting of five plates and 240 figures in the text, are very helpful, and the entire treatise is a modern, compact, and scientific exposition of present methods used in diagnosticating disease. The student should make it his companion, and the practising physician should refer to it with great frequency.

**Abdominal Operations.** By **B. G. A. Moynihan,** M.S. (London), Senior Assistant Surgeon to Leeds General Infirmary, England. Octavo, 741 pages, with 701 original illustrations, many in colors. Philadelphia and London: W. B. Saunders & Company. 1905. (Price, Cloth, $7.00 net.)

This instructive book will be read with interest by every abdominal operator, especially those who pay particular attention to the gastrointestinal tract. Its chief value is in the technic it inculcates relating to preparation of the patient for operation, the conduct of the operation itself and the after-treatment; also, as relates to those operations which the author classifies as common to both sexes.

Mr. Moynihan is an experienced surgeon and whatever he says is entitled to the weight of authority. The operative treatment of acute peritonitis, perforating gastric ulcer, chronic gastric ulcer, and cancer of the stomach will attract attention. The technic of all operations on the stomach is described and illustrated with great clearness. In his intestinal surgery Moynihan ignores buttons and all other mechanical contrivances, relying entirely on the suture in some form to unite the segments of the bowel. He leans with favor toward the Connell suture, describing it in great detail and devoting eight illustrations to its elaboration.

The author presents his own experience in lucid fashion and quotes cases in support of his opinions, not only from his own practice but also from that of other surgeons of experience. The work is beautifully illustrated in large part from original drawings and is printed on heavy book paper, making it at once a handsome as well as a durable volume. It is a book which every abdominal surgeon will need, and its guidance will be found of value in many difficult conditions.

**The National Standard Dispensatory.** Containing the Natural History, Chemistry, Pharmacy, Actions and Uses of Medicines, including those recognised in the Pharmacopeias of the United States, Great Britain and Germany, with numerous references to other foreign pharmacopeias. In accordance with the United States Pharmacopeia, 8th decennial revision of 1905 by authorisation of the Convention. By **Hobart Amory Hare,** B.Sc., M.D., Professor of Theraputics in the Jefferson Medical College, Philadelphia, Member of the Committee of Revision of the U. S. P.; **Charles Caspari, Jr.,** Ph.G., Phar.D., Professor of Pharmacy in the Maryland College of Pharmacy, Baltimore, Member of the Committee of Revision of the U. S. P.; and **Henry H. Rusby,** M.D., Professor of Botany and Materia Medica in the College of Pharmacy of the City of New York, Member of the Committee of Revision of the U. S. P. Imperial octavo, 1858 pages, 478 engravings. Cloth, $7.25 net; leather, $8.00 net. Thumb index, $.50 extra. Lea Brothers & Co., Publishers, Philadelphia and New York. 1905.

The National Dispensatory is a work needed in every physician's office where medicines are dispensed, and it is indispensable in the pharmacy, where reference must be made constantly to the preparation of drugs used in presciptions. The old United States Dispensatory was studied by every office student as a part of his preparatory work. It was an orthodox exponent of the pharmacy of the period.

This work, built on a more comprehensive scale, takes the place of the former and should be accessable to physicians and pharmacists as a reference volume.

The tripartite authorship of Hare, Rusby, and Caspari, stands in the place of Wood and Bache who for so many years were the recognised authority on dispensatory questions. This new work contains authority to use for comment the eighth revision of the U. S. Pharmacopeia lately issued; indeed, these authors are all members of the committee of revision. The publishers' descriptive comments relating to the part each author plays, and the supplementary make-up of the book are pertinent.

Of its authors, Dr. Rusby has treated the department of pharmacognosy, including the minor as well as the major drugs of the entire globe, a service never before rendered; Prof. Caspari deals with pharmacy, giving full information regarding methods and products, with descriptions and explanations of the most approved apparatus and tests, and Dr. Hare has written the section on medical action and uses, giving a direct and compact presentation of modern theraputics. An appendix of 60 pages contains all necessary tables, formulas, tests, etc., for practical use. The general index, of about 90 pages, contains full reference, to every page in the text, making it a repertory of the world's knowledge of drugs, and the therapeutical index, of about 40 pages, contains, under the name of each disease, references to all the medicines employed in its treatment, leading the reader to the points in the text where the conditions indicating their employment and choice will be found. In a word, the National

Standard Dispensatory is a new, practical and authoritative work containing information on all substances used in medicine and pharmacy at the present day. The volume is embellished with no fewer than 478 new and instructive engravings in the text.

We deem it fortunate that the authorship and publication of the dispensatory have fallen into such competent and conscientious hands.

**The Diagnostics of Internal Medicine.** By **Glentworth Reeve Butler,** M.D., Attending Physician to the Brooklyn Hospital. Octavo, pp. xxxiv-1168. With five colored plates and 288 illustrations and charts in the text. Second edition. New York and London: D. Appleton & Company. 1905. (Price, $6.00.)

The appearance of a second edition of this valuable work furnishes the occasion for a repitition of the commendation we bestowed upon the book in this JOURNAL (January, 1902. p. 455) when the first edition was published. The author has adhered to his original plan in the republication of his book, which is wise, as the arrangement and classification have proved satisfactory. Some changes have become necessary by reason of advances in medical science and some additions to the illustrations have been made. As at present constituted the work represents the most advanced thought concerning the diagnosis of internal disease, and is a credit to the distinguished author. He still persists in "tumour, colour," and the like, which we consider a blemish on an otherwise nearly perfect treatise.

**A Manual of Clinical Chemistry, Microscopy and Bacteriology.** By **M. Klopstock** and **A. Kowarsky,** Berlin. Translated by Thew Wright, M.D., 12mo, pp. 296. Illustrated. New York: Rebman Company. 1905. (Price, $2.25 net.)

We regard this as one of the best books of its class. A small manual, setting forth the essentials of microscopy, bacteriology and clinical chemistry, adapted to the needs of the general practitioner of medicine. It has been faithfully translated, giving with a smoothness of diction the plain facts of the original. We commend it also to the student, though it is not intended to take the place of larger textbooks which he must study as a matter of course. The illustrations, seventy in number, thirty of which are printed in colors, deserve commendation.

**Color-Vision and Color-Blindness.** A Practical Manual for Rail-Road Surgeons. By **J. Ellis Jennings,** M.D. (University of Pennsylvania). Formerly Clinical Assistant Royal London Ophthalmic Hospital; Professor of Diseases of the Eye, Medical Department Barnes University, St. Louis. Second edition. Revised with illustrations. Pp. 132. Small octavo. Philadelphia: F. A. Davis Company. 1905. (Price, $1.00 net)).

This book, though prepared especially for railway surgeons, is of use to every physician interested in ophthalmology. It is also of particular value to every examining physician or surgeon for civil service, pensions, and the like. It is nine years since the first edition was published, hence a revision became necessary. Two new chapters have been added; also a description of Williams's and Thompson's lanterns, Williams's and Black's semaphore charts, Abney's pellet test for central scotoma, and five new llustrations. The book is an expression of all of value on this subject, and is without a rival.

**The Principles and Practice of Medicine.** By **William Osler,** M.D., Honorary Professor of Medicine, Johns Hopkins University, Baltimore. Sixth edition. Octavo, pp. 1161. New York and London: D. Appleton & Company. 1905.

During the past fourteen years this treatise has been a favorite with both students and practitioners of medicine. We have heretofore expressed our views of the work in considerable detail, and need not repeat them now. It is, however, in keeping with the proprieties of the occasion for us to reaffirm them, and to add that this edition is the most complete of any of its predecessors.

Many chapters have been rewritten, every chapter in the work has been thoroughly revised, and much new material has been added. In this revision special attention has been given to new points in treatment. Though much matter has been added, an enlarged page and a different type have enabled the publishers to keep the book within the limits of a single, compact volume,—a desideratum of no mean importance.

**Manual of the Diseases of the Eye.** For Students and Practitioners. By **Charles H. Hay,** M.D., Chief of Clinic and Instructor in Ophthamology, College of Physicians and Surgeons, Medical Department of Columbia University, New York, 1890-1903; Ophthalmic Surgeon to the City Hospital, Randall's Island, New York, etc., etc. Fourth edition, revised, with 360 illustrations, including 21 plates and 60 colored figures. Small octavo, pp. viii.-391. New York: William Wood & Co. 1905. (Price, $2.00 net).

This popular manual appeared in its first edition in 1900, since which time three more editions have been called for, besides several reprints. No higher testimony of the value of the book could be presented, and the author cannot but be gratified at such an appreciation of his labors. This fourth edition has been revised with care and many additions have been made. It is emphatically a book for general practitioner and student. It is profusely illustrated, no less than sixty figures being in colors. It is easily one of the most satisfactory manuals on the eye in print and justly deserves the large sale which it is having.

**The Physicians' Visiting List** (Lindsay & Blakiston's) for 1906. Fifty-fifth year of its publication. Sold by all booksellers.

This veteran handy pocket visiting list is always acceptable. It is compact, handsome, and useful. Though the oldest book of its kind in the field, it is ever young, being brought forward each year to meet changes and improvements. The dose-table in this edition has been revised in accordance with the pharmacopeia of 1900, lately issued. P. Blakiston's Son & Company, Philadelphia, are the publishers.

**The Medical Record Visiting List or Physicians' Diary**, for 1906. New York. William Wood & Company Medical Publishers.

This substantial diary has been among the favorites for a long time. It retains its general form but some material not necessary for an emergency reference has been eliminated, while some has been added to make it more useful for that purpose. It is well adapted to a busy practice and is made in several styles to meet the wants of different physicians, specialists or generalists, those with large or moderate practices as the case may be. The prices vary between $1.25 and $4.00, according to size and kind of binding.

**The Practitioners' Visiting List** (Heretofore known as the Medical News Visiting List) for 1906. An invaluable, pocket-sized book, containing memoranda and data important for every physician, and ruled blanks for recording every detail of practice. The Weekly, Monthly and 30-Patient Perpetual contain 32 pages of data and 160 pages of classified blanks. The 60-Patient Perpetual consists of 256 pages of blanks alone. Each in one wallet-shaped book, bound in flexible leather, with flap and pocket, pencil and rubber, and calendar for two years, $1.25. Thumb-letter index, 25 cents extra. By mail, postpaid to any address. Descriptive circular showing the several styles sent on request. Lea Brothers & Co., Publishers, Philadelphia and New York, 1905.

The foregoing title is sufficiently descriptive, hence little need be added. The text has been revised, making it conform to the latest thought, and bringing it forward to the immediate present. It contains among other valuable information a scheme of dentition; tables of weights and measures and comparative scales; instructions for examining the urine; table of eruptive fevers; incompatibles, poisons and antidotes; directions for effecting artificial respiration; extensive table of doses; an alphabetical table of diseases and their remedies and directions for ligation of arteries. The record portion contains ruled blanks of various kinds, adapted for noting all details of practice and professional business. As a physician's clinical diary it ranks among the best.

